# Psychological and neuropsychological correlates of dependence-related behaviour in Medication Overuse Headaches: a one year follow-up study

**DOI:** 10.1186/1129-2377-14-59

**Published:** 2013-07-04

**Authors:** Françoise Radat, Sandra Chanraud, Georges Di Scala, Virginie Dousset, Michèle Allard

**Affiliations:** 1 Department of Treatment of Chronic Pain Patients, Pellegrin University Teaching Hospital, Bordeaux, France; 2 CNRS, INCIA UMR 5287, Bordeaux University, F-33400, Talence, France; 3 EPHE, Bordeaux, France; 4 Centre de Traitement et d’Evaluation de la Douleur, CHU Pellegrin, 33076, Bordeaux cedex, France

**Keywords:** Medication overuse headache, Dependence-related behaviour, Prospective, Iowa gambling task

## Abstract

**Background:**

Medication Overuse Headache (MOH) can be related in some patients to dependence-related behaviour characterised by craving, a deficit in controlling substance intake, which is associated to orbitofrontal cortex (OFC) dysfunction. The aim of this study was to explore the psychological correlates in MOH patients and the functioning of the OFC through neuropsychological assessment (Iowa Gambling Task: IGT) and to relate it to prognosis at a one year follow-up point.

**Findings:**

Seventeen subjects suffering from probable MOH were included and compared to 19 migraineurs and to 17 controls. The results show significant between group differences for behavioural dependence, depression, anxiety, catastrophizing. There were no between group differences for impulsivity. Mean IGT score did not allow differentiation of MOH patients from the other groups, whereas the score was significantly different between opiate abusers and other medication abusers (45 +/−5.7 versus 57.1 +/−8.2, p = 0.019). Among the clinical variables rated at inclusion, the amount of acute headache medication taken per month was the only one predicting the prognosis (RR = 1.05, 95% CI = 1-1.06, p = 0.04). A slight increase in risk of relapse at 1 year was observed in patients with poorer IGT scores (RR = 0.92, 95% CI = 0.85-1, p = 0.05) and higher behavioural-dependence scores (RR = 1.07, 95% CI = 1–1.14, p = 0.05). None of the other psychological variables predicted relapse risk.

**Conclusions:**

These results must be interpreted with caution due to the low number of subjects. They showed a deficit in decision making processes in MOH patients who overuse medications containing psychoactive substances like opiates. Moreover dependence-related variables are related to the prognosis.

## Introduction

The role of behavioural disorders and particularly an addictive component of behaviour in Medication Overuse Headaches (MOH) is a matter of debate [1]. Orbitofrontal dysfunction is known to be at work in addictive behaviour. Indeed, orbitofrontal cortex is involved in the ability to inhibit craving behaviour through decision making impairment [2,3]. Other psychological dimensions may be implicated in dependence related behaviour in MOH patients. In particular, MOH patients have been shown to be at risk of depressive disorders and anxiety disorders when compared to episodic migraine patients [4]. Cupini and al. [5] emphasized the presence of obsessive compulsive disorders in these patients as part of the compulsive drug-seeking behaviour. In a one year follow-up study anxiety and depression were not reliable prognosis factors for MOH, in contrast to addictive behaviour [6]. Nevertheless, no study has focused on catastrophizing in MOH patients or on impulsivity while those dimensions may be of interest in determining dependence related behaviour.

Our objective was therefore to compare anxiety, depression, catastrophizing and impulsivity dyscontrol as well as decision-making impairment in MOH patients, episodic migraine patients and healthy controls. Then, a follow up was performed in order to relate psychological and neuropsychological variables to prognosis.

## Methods

This is the clinical part of an imaging study implying rest fMRI and ^18^F FDG PET. Results of the imaging study will be presented elsewhere. The study was prospective, non-randomised, comparing MOH evolving from migraine patients to episodic migraine sufferers (EM) and to healthy volunteers without headache (HV). MOH and EM patients have been followed up over one year. Patients asking for a consultation at the neurological department at the University Hospital in Bordeaux were included if they fulfilled migraine diagnostic criteria (ICHD-II 1.1 and 1.2) or probable MOH diagnostic criteria (ICHD-II 8.2) with prior migraine and if they were 18 years old or more. A prior episode of MOH was an exclusion criteria for the migraine group The healthy volunteers were recruited among Bordeaux University Hospital staff members.

Clinical data collection comprised a standardized interview process inquiring for crisis frequency and medication intake frequency during the last three months and acute and prophylactic antimigraine drugs currently used. Patients from the EM and MOH groups were given a headache diary, recording days with headache and acute headache medication intake during the follow-up period. All subjects answered self-administered questionnaires. In order to assess depression, the Beck Depression Inventory ((BDI-13 items) [7] was used. Anxiety was assessed using the State Trait Anxiety Inventory (STAI-state) [8]. Catastrophizing was assessed using the Pain Catastrophizing Scale (PCS) [9]. The Barratt Impulsiveness Scale (BIS- 11) [10] is a self-report measure of impulsive personality traits. The Medication Dependence Questionnaire for Headache sufferers (MDQ-H) [11] is a rating scale assessing dependence to medication in headache patients. Then, all subjects underwent IGT [12], a computerised task which is a test aiming to detect decision-making impairment. It is a test where the subject is confronted with a decision that involves a conflict between an immediate reward and a long term negative consequence. The lower the score achieved, the poorer the decision-making performance is.

Subjects from the MOH group underwent either an inpatient or outpatient withdrawal procedure. MOH and EM groups were re-examined at 12 months from inclusion. Patients from the MOH group were classified in a binary way as relapse or non relapse depending on whether they fulfilled ICHD-II criteria for MOH at month twelve visit.

Statistical analysis was performed using SPSS version 17.0 software. Descriptive analysis was performed and the differences between groups were tested using chi square and a one-way analysis of variance when appropriate. In order to compare the score results obtained by the questionnaires and the IGT between the three groups a one-way analysis of variance was also used, and t-test was used to compare groups in pairs. Logistic regression analysis was performed in order to predict relapse within one year. For the patients that have been followed up during one year, scores evolution was tested using paired t-test.

## Findings

Fifty-one subjects were included in the study: 17 patients suffering from MOH, 19 EM 17 HV.

Sociodemographic characteristics of participants are presented in Table 1.The three groups were comparable for sex, age, family situation and work status.

**Table 1:** Socio-demographic and clinical data within groups

	**N = 17**	**N = 19**	**N = 17**
	**MOH group**	**EM group**	**HC group**
Gender			
Male	4	5	4
Female	13	14	13
Age (years)	46.3 +/−10.7	46 +/−10	46.5 +/−10.5
Age of 1st occurrence of migraine	17 +/−9	19.5 +/−12	-
Number of day with headache/month	23.5 +/−7.1	2.5 +/−1.1	-
Number of tablets of acute headache medication/month	82 [360–21]	6.2[16−1]	-

The mean number of days with headaches was 23.5 +/− 7.1 in the MOH group and 2.5 +/−1.1 in the EM group. The mean duration of MOH was 6.8 years; duration ranged from 4 months to 30 years. The acute headache medication overused by MOH patients are shown in Table 2. Five patients were overusing either opiates or a combination of analgesics containing opiates derivates. The total amount of acute headache medication taken per month was 82 [360–21] in the MOH group and 6.2 [1] in the EM group.

**Table 2:** Acute headache medication overused by MOH patients

**ICHD 2 diagnosis**	**n**
Triptan (ICHD-2: 8.2.2)	4
Analgesic (ICHD-2: 8.2.3)	2
Opioid (ICHD 2: 8.2.4)	2
Combination analgesic (ICHD 2: 8.2.5)	1
Combination of medication (ICHD 2:8.2.6)	8
Triptan	7
Analgesic	4
Combination	3
Opioid	1

**Figure 1:**
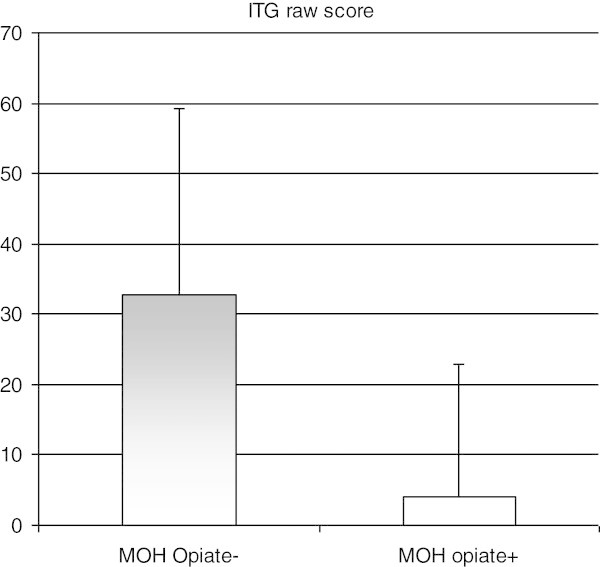
IGT row score in opiate abusers and non opiate abusers in the MOH group.

The mean scores obtained in each questionnaire assessing the psychological dimension in each groups are presented in Table 3. The results show that mean scores were higher in MOH than in EM and HV for dependence (MDQ-H), depression (BDI), anxiety (STAI), catastrophizing (PCS). The between groups analysis of variance for anxiety (STAI)) and depression (BDI) indicated a significant statistical group effect (F = 5,7, p = 0;006 ; F = 6;3, p = 0;003). On the contrary, there were no between group differences for impulsivity (BIS). Total raw scores for IGT did not allow to differentiate MOH patients (IGT total raw score score = 26.2+/−24.8) from episodic migraineurs (29.0+/−29.7) nor from volunteers without headache (14.7 +/−30.4) whereas within the MOH group there was a significant difference between opiate abusers and other medication abusers (4+/−18.9 versus 32.8 +/−26.5, p = 0.028) (Figure 1). When comparing MOH patients to EM patients, only MDQ-H and PCS scores were significantly different, whereas when comparing MOH to HV, MDQ-H, PCS, BDI and STAI scores were significantly different (Table 2).

**Table 3:** MDH-Q, BDI, STAI, PCS, BIS and IGT scores within groups

	**MOH, n = 17**	**EM, n = 19**	**HV, n = 17**	**F, p**
MDH-Q	85.2 +/−24 [47–133]*§	35.3 +/−12.6 [9–63]°	21.7+/−1.9 [21–29]	F = 78.6, p < 0.000
BDI	5.2 +/−4.2 [0–12]§§	3.5 +/−3.9 [0–13] °°	1.0+/−1.3 [0–4]	F = 6.3, p = 0.003
STAI	40.5 +/−10.4 [22–56]§§§	35.8 +/−9.8 [20–60]°°°	29.4+/−8.5[21–53]	F = 5.7, p = 0.006
PCS	28.3 +/−12.5 [10–51]**§	20.3 +/−12 [0–38]∞	4.8+/−6 [0–51]	F = 21.3, p < 0.000
BIS	58 +/−7.7 [40–73]	57.2+/−9.8 [42–85]	59 +/−6.7 [51–74]	F = 0.226, NS
IGT total raw score	26.2 +/−24.8	29.5 +/−29.7	14.7 +/−30.4	F = 1.25, NS

MOH vs EM: *:p < 0.000, **:p = 0.05.

MOH vs HV: § p < 0.000, §§ p = 0.001, §§§p = 0.002.

EM vs HV: ° p < 0.000, °° p = 0.01, °°° p = 0.04.

Among the clinical variables rated at inclusion, the number of acute headache medication units taken per month was the only one predicting the prognosis (RR = 1.05, 95% CI = 1-1.06, p = 0.04). A slight increase in risk of relapse at 1 year was observed in patients with poorer IGT raw scores (RR = 0.92, 95% CI = 0.85-1, p = 0.05) and higher MDQ-H scores (RR = 1.07, 95% CI = 1–1.14, p = 0.05). None of the other psychological variables predicted relapse risk.

For the MOH group a one year a significant decrease was observed for MDQ-H (t = 5,7, p < 0,000) and for PCS (t = 3,7, p = 0,003) and for the EM groups no significant differences were observed.

## Discussion

To summarise, this study showed between group differences for anxiety, depression, catastrophizing and behavioural dependence but not for impulse dyscontrol and decision-making impairment. However, MOH with opioid medication overuse exhibited a significant disturbance in decision making when compared with MOH overusing other acute headache medication. Moreover dependence related behaviour and decision-making impairment at inclusion weakly predicted one year prognosis in MOH whereas other variables did not.

Catastrophizing has been reported to be associated with a higher risk of prescription opioid abuse in pain patients [13,14]. In the field of headaches, it has been showed that subjects with chronic daily headaches exhibit increased catastrophizing scores when compared to episodic migraineurs, higher catastrophizing being associated with lower quality of life [15]. Moreover the prevention of MOH in high frequency migraine patients by a cognitive behavioural program is associated with a lowering of catastrophizing [16].

There are a few studies examining psychological factors as predictors of prognosis in MOH patients. The Akershus study of chronic headache showed that Dependence scores (SDS) predict 2 to 3 year prognosis after detoxification [17]. Smoking, alcohol consumption and the doses of antimigraine drugs used allowed to predict one year follow-up relapse in detoxified MOH but not in anxiety disorders or affective disorders [6]. In a one year follow-up study of 72 subjects with MOH from the general population Fontanillas showed that opioid abuse was associated with a bad prognosis [18]. In this direction, our results confirm that the dependence related behaviour is a stronger predictor of prognosis than emotional disturbances. Indeed, despite the small size of the sample, the amount of acute headache medication taken and the MDQ-H score at inclusion significantly predicted prognosis at one year whereas all emotional measures did not.

The IGT allows to measure the ability to defer immediate reward, taking into account the long term negative consequences, and it is altered in prefrontal-damaged patients. In a sample of MOH patients, Biagianti and al [19] showed a statistically significant deficit in IGT net score compared to healthy matched controls. Patients were retested one year later and IGT performance remained negative. The IGT score did not allow to predict relapse in medication overuse after detoxification [20]. In a similar vein, Gomez-Beldarrain evaluated orbito-frontal impairment using other neuropsychological tasks. They found a significant impairment in the orbito-frontal tasks performance in MOH compared with controls. Moreover, in the one year follow-up this performance predicted relapse in MOH. Our study failed to demonstrate any difference between MOH and the two controls groups despite a slightly lower performance in MOH than in episodic migraineurs. Our healthy control sample clearly exhibited a lack of motivation when performing the task. On the other hand, the comparison between opiate overusers and other medication overusers showed a significant difference between the two groups on the IGT total net score. This result can be put into parallel with the fact that in Fumal’s sample of MOH patients, the hypofunctioning of the orbito-frontal cortex showed with PET was due to the subsample of patients overusing opiates [21]. Among the MOH sample, the IGT total net score predicted the prognosis at a one year follow up visit confirming the previously cited studies.

The strength of this study is its one year prospective design, the assessment of rarely explored psychological dimensions such as impulsivity and catastrophizing. The major weaknesses are the small number of subjects and the motivation failure in the healthy controls, thereby it would be very usefull to present further resuklts with more subjects. Nevertheless the study allows to confirm the utmost importance of dependence related behavioural disturbance in characterising MOH patients. Moreover it seems determinant for prognosis in an illness characterised by its poor prognosis. These results lead us to advise strong management of the psychological dimension in MOH patients. Cognitive behavioural therapies in particular can address catastrophizing, emotional disorders and impulse dyscontrol and motivational interviewing should be part of the management of the dependence related behaviours.

## Abbreviations

MOH: Medication overuse headache; EM: Episodic migraine; HV: Healthy volunteer; ICHD-II: International classification of headache diagnosis 2nd version; BDI: Beck depression inventory; STAI: State and trait anxiety Inventory; PCS: Pain catastrophizing scale; BIS: Barrat impulsivity scale; MDQ-H: Medication dependence questionnaire in headache; OFC: Orbito frontal cortex; IGT: Iowa gambling task; PET: Positon emission tomography.

## Competing interests

FR: Lilly France, UPSA, Pfizer.

SC, GD, VD, MA: The authors declare that they have no competing interests.

## Authors’ contribution

FR: conception and design, acquisition of data, statistical analysis, interpretation of data and discussion of results, drafting the article, final approval of the complete manuscript. SC: interpretation of data and discussion of results, final approval of the complete manuscript. G DS: interpretation of data and discussion of results, final approval of the complete manuscript. VD: acquisition of data, discussion of results, final approval of the complete manuscript.MA: conception and design, interpretation of data and discussion of results, final approval of the complete manuscript. All authors read and approved the final manuscript.
